# Smartphone Addiction as a Mediating Pathway Between Personality Traits and Job Performance

**DOI:** 10.1192/j.eurpsy.2026.10831

**Published:** 2026-07-31

**Authors:** N. Rmadi, A. Kchaou, R. Affes, A. Hrairi, M. Hajjaji, K. Jmal Hammami

**Affiliations:** 1 Occupational medicine department, Hedi Chaker university hospital; 2 Family medicine department, Faculty of medicine of sfax, Sfax, Tunisia

## Abstract

**Introduction:**

Personality traits influence professional performance, yet their impact may be distorted by behavioral addictions.

**Objectives:**

To determine whether smartphone addiction mediates the relationship between personality traits and work performance among medical residents.

**Methods:**

A cross-sectional study was conducted in 2025 among 429 Tunisian medical residents. Personality was assessed with the BFI-20, smartphone addiction with SAS-SV, and performance with IWPQ. Mediation models using hierarchical linear regression estimated indirect effects.

**Results:**

Conscientiousness was a strong positive predictor of job performance (β=0.488, p<0.001). However, smartphone addiction significantly mediated the effects of extraversion (indirect effect β=–0.219, p<0.001) and neuroticism (β=–0.161, p<0.001) on performance. While these traits were directly associated with higher performance, their benefits were partly offset when residents exhibited problematic smartphone use.

**Conclusions:**

Smartphone addiction acts as a mediating pathway, attenuating the positive effects of certain personality traits on performance. Targeted interventions to reduce smartphone overuse could preserve the beneficial influence of personality on residents’ occupational efficiency.

**Disclosure of Interest:**

None Declared

